# Supplementary material for: Serum zinc level and hepatic fibrosis in patients with nonalcoholic fatty liver disease
Source: PLoS One. 2020 Oct 23;15(10):e0240195. doi: 10.1371/journal.pone.0240195 (PMC7584204; doi:10.1371/journal.pone.0240195)
Supplement: Attachment — Submitted filename: Respone to reviewers.docx [file pone.0240195.s001.docx]

Reviewer #1: Kim MC have described the manuscript entitled “Serum zinc level and hepatic fibrosis in patients with nonalcoholic fatty liver disease”. The authors demonstrated the relationship between serum Zn level and hepatic fibrosis in NAFLD. Zn is reported to be important nutritional factor related to liver fibrosis and various types of carcinogenesis, including HCC. However, this article has some critical problems and limitations. Specific comments are given below.

1. Regarding serum Zn levels in NAFLD, recent reports have already shown the relationship between Zinc and NAFLD, and prognosis in biopsy-proven NAFLD cohort (EJGH2019, Nutrition and Cancer 2019). In this report, NAFLD participants were diagnosed with NAFLD liver fat score, not biopsy. And also, they only used Fib4 index for evaluating liver fibrosis. Liver biopsy is the gold standard for diagnosing NAFLD and assessing liver fibrosis, therefore, this study has a lack of novelty for publication.

: We appreciate your valuable comment. As you commented, the gold standard method for the diagnosis of NAFLD and liver fibrosis is the evaluation of histological findings by liver biopsy. However, it is not widely used because of the invasiveness and the cost. This study was conducted based on a nationally representative survey complex, stratified, multistage, and probability cluster sampling of the noninstitutionalized population (the Korea National Health and Nutritional Examination Survey 2010). A total of 8,958 participants completed the survey. Therefore, it is hard to check liver biopsy for the diagnosis of NAFLD and hepatic fibrosis in these many participants. In this study, we evaluated NAFLD (NAFLD liver fat score) and hepatic fibrosis (FIB-4) using commonly used blood tests. The diagnostic accuracy of NAFLD liver fat score for NAFLD is that AUROC was 0.86-0.87 with sensitivity 86% and specificity 71% (Nat Rev Gastroenterol Hepatol 2018;15(8):461-478). Significant hepatic fibrosis was defined as FIB-4 index >1.3 (Hepatology. 2006;43(6):1317-25). This study may be helpful to detect NAFLD and to assess hepatic fibrosis with commonly used blood tests in the settings of large scaled survey such as a population-based study. We described this limitation in the discussion of the manuscript.

(page 14, line 12)

“First, this study did not consider the histologic findings of hepatic steatosis and fibrosis in patients with NAFLD. We assessed hepatic steatosis and fibrosis using NAFLD liver fat score and FIB-4 index. The gold standard method for the diagnosis of NAFLD and liver fibrosis is the evaluation of histological findings by liver biopsy. However, it is not widely used because of the invasiveness and the cost. This study was conducted based on a nationally representative survey complex, stratified, multistage, and probability cluster sampling of the noninstitutionalized population (the KNHANES 2010). A total of 8,958 participants completed the survey. Therefore, it is hard to check liver biopsy for the diagnosis of NAFLD and hepatic fibrosis in these many participants. In this study, we evaluated NAFLD (NAFLD liver fat score) and hepatic fibrosis (FIB-4) using commonly used blood tests. The diagnostic accuracy of NAFLD liver fat score for NAFLD is that AUROC was 0.86-0.87 with sensitivity 86% and specificity 71%.[32]”

2. The authors have set the cut off value of Fib4 index as 1.3 for evaluating significant fibrosis. As the authors have described as limitation in Discussion part, Fib4 index of 1.3 is known as the cut off point for rule out the patients with mild/no fibrosis, and the proportion of the patients with Fib4 of 2.67 or more, which is focused more for the cut off with positive predictive value, was only 8%. Fibrosis is well known as the strong factor for predicting prognosis for NAFLD patients, so the cohort in this study was not good enough, because the number of patients with significant fibrosis, which reach the level to affect the prognosis might be small, and this results less information for clinical practice.

: We agree with your comment. Your comment was also our concern of the analysis in this study. In this study, we defined the cut-off point of FIB-4 for significantly liver fibrosis as 1.3. The reason is as follows. A meta-analysis reported that the cut-off point of FIB-4 for advanced fibrosis in NAFLD was 2.67 or 3.25 (Hepatology. 2017;66(5):1486-1501) while another meta-analysis showed that the cut-off point was 1.3 (Hepatol Res. 2016;46(9):862-70). The cut-off point of 2.67 had an advantage in terms of positive predictive value for identifying advanced fibrosis in NAFLD while that of 1.3 had a 90% negative predictive value (Clin Gastroenterol Hepatol. 2009;7:1104-12). In the present study, the number of patients with FIB-4 >2.67 was 8 (3%) while 62 patients (21%) had the level of FIB-4 >1.3. It is difficult to analyze the univariable and multivariable logistic regression analysis using FIB-4 cutoff with 2.67. Thus, we defined FIB-4 cutoff as 1.3 (Hepatol Res. 2016;46(9):862-70) although the ideal FIB-4 cutoff was 2.67 or 3.25 (Hepatology. 2017;66(5):1486-1501). This is the limitation of our study. We described this limitation in the discussion of the manuscript.

(page 14, line 22)

” The diagnostic accuracy of the FIB-4 index for advanced fibrosis in histology is high in patients with NAFLD (mean value of area under curve, 0.80; 95% CI, 0.77–0.84) [32]. In this study, we defined the cut-off point of FIB-4 for liver fibrosis as 1.3. The reason is as follows. A meta-analysis reported that the cut-off point of FIB-4 for advanced fibrosis in NAFLD was 2.67 or 3.25 while another meta-analysis showed that the cut-off point was 1.3 [33, 34]. The cut-off point of 2.67 had an advantage in terms of positive predictive value for identifying advanced fibrosis in NAFLD while that of 1.3 had a 90% negative predictive value [35]. In the present study, the number of patients with FIB-4 >2.67 was 8 (3%) while 62 patients (21%) had the level of FIB-4 >1.3. It was difficult to perform the univariable and multivariable logistic regression analysis using the FIB-4 cut-off with 2.67. Thus, we defined the cut-off point of FIB-4 as 1.3 in this study [34].”

3. How did author set the cut-off value of serum zinc level? If the authors emphasize the importance of Zn level in NAFLD, they should use the definition on Zn deficiency. Zinc deficiency is defined as serum Zn level <70 μg/dL based on previous report (Harrison’s Principles of Internal Medicine 2015). Otherwise, clinical impact should be small.

: We agree with your comment. The rationale for the 140 µg/dL as the cut-off for Zinc level is as follows. In our study, the median value of zinc was 135 µg/dL. Thus, we regarded the patients with zinc level ≥140 µg/dL (54 percentile) as the high level of serum zinc and those with zinc level <140 µg/dL as the low level of serum zinc. When 135 µg/dL of zinc level was applied for cut-off in this study, the results showed a similar tendency compared with 140 µg/dL cut-off (data not shown). We totally understand your comment about the confusion of low or high Zn level. In this study we did not represent ‘zinc deficiency’ or ‘hypozincemia’. The low and high Zn level in this study means relative concepts which show the trend of decreased or increased Zn level in KNHANES cohort. (high Zn level (≥140 µg/dL) and low Zn level (<140 µg/dL))

4. Following the above, mean value of serum Zn in whole participants was 139.8 μg/dL. This value is within normal limits or higher, and it is expected that the majority of participants in this study has normal liver without any hepatic inflammation and fibrosis.

: Thank you for your comment. Please check the above answer to question #3.

5. The mechanism to induce lower Zn levels in NAFLD in this study was unclear. Hypoalbuminemia is one of the most important factors that can lead to Zn deficiency because Zn mainly binds to albumin during transportation in the blood. Additionally, hypoalbuminemia is a common metabolic disorder induced by liver cirrhosis. The authors need to add the information of serum albumin in Table1, and put albumin in logistic regression analysis.

: Thank you for this comment. As we mentioned introduction and discussion section, the mechanisms of induced lower zinc level in NAFLD is bi-directional. The dietary habit of Zn intake affects the progression of NAFLD, and mineral metabolism abnormalities due to NASH/cirrhosis can affect zinc levels. This study was conducted based on the data from the KNHANES, a nationally representative survey, is performed by the Korea Centers for Disease Control and Prevention (KCDC). However, the albumin level and dietary amount of Zn were not provided in KNHANES data. Just as in our study, significant liver fibrosis (FIB-4 index ≥1.3) was observed in 62 patients (21%). We agree with your comment for the relationship between serum zinc and albumin. To find the mechanism to induced lower Zn levels in NAFLD has a limitation in our study. We mentioned this point in revised Discussion section:

(page 15, line 22)

“Third, we were unable to determine the oral intake of Zn and serum albumin level which could affected to the lower Zn in NAFLD because the data from the KNHANES did not provide information about Zn intake and albumin. We assumed that the patients with relatively low Zn levels were more likely to have NAFLD or NASH. However, this study could not evaluate the mechanism of lower zinc level in patients with NAFLD affected by Zn intake or albumin level. Zinc-rich foods are meat, shellfish, beans, seeds, nuts, dairy foods, eggs, and whole grains. Although the KNHANES provided information about the intake of these foods, it did not provide information about oral Zn intake.”

6. Circadian variation of Zn levels has been recognized well. Were the blood samples of participants in this study collected under the same conditions, such as in the morning with a fasting state?

: Blood samples of participants collected under the same conditions in day time with a 12h of fasting. We mentioned this point in The Method section as follows:

(page6, line 4-21)

“Blood tests, including ~ were conducted after 12 h of fast. All health examination components including blood sample collection have been conducted in the Mobile Examination Centers (MECs) that travel to each survey location. These MECs provide a standardized environment and equipment. ~ All blood samples were analyzed within 24 h after arrival at the Central Testing Institute.”

7. To conduct the linear regression analysis, the authors should weigh the R-values more compared with P-values. The authors showed the significant relationship in P-value (p=0.038) with Pearson correlation test, but coefficient value was -0.120. This result indicated very weak correlation on these two factors, And the statistical interpretation is not strong.

: We appreciate your valuable comment. According to your comment, we re-analyzed the correlation between serum zinc and FIB-4 index adjusting potential confounding factors (gender, BMI, r-GTP, fasting glucose level, triglyceride, LDL cholesterol, HDL cholesterol, diabetes mellitus, and vitamin D level). The partial correlation coefficients (*r*) adjusted for gender, BMI, r-GTP, fasting glucose level, triglyceride, LDL cholesterol, HDL cholesterol, diabetes mellitus, and vitamin D level between serum zinc level and FIB-4 index is *r*= -0.177 with P=0.003. We added these findings in the methods and results sections of the revised manuscript.

(page 7, line 7) – methods section

“Partial correlation analysis between serum Zn level and FIB-4 index was done adjusting potential confounding factors which could affect the relationship.”

(page 9, line 12) – results section

“We analyzed the correlation between serum zinc and FIB-4 index adjusting potential confounding factors. The partial correlation coefficients (r) adjusted for gender, BMI, GGT, fasting glucose level, triglyceride, LDL cholesterol, HDL cholesterol, diabetes mellitus, and vitamin D level between serum zinc level and FIB-4 index is *r*= -0.177 with *p*=0.003.”

Reviewer #2: Data from a large cohort.

Limitations were partially described by the authors.

Remarks:

-only use of non invasive marker FIB4? The cutt-off used in this trial was 1.3 for significant fibrosis. Is age also taken into account?

: We appreciate your valuable comment. The survey of this study did not include imaging based elastography such as transient elastography. As you commented, we calculated non-invasive fibrosis markers besides FIB-4 index: (i) AST to Platelet Ratio (ARPI) score =100× (AST/upper limit of normal)/platelet count (×10^9^/L) (Hepatology, 2003 Aug;38(2):518-26); (ii) BARD score = (BMI ≥28 = 1 point, AST to ALT ratio ≥ 0.8 = 2 points, diabetes = 1 point), scale 0–4 (Gut 2008;57:1441–1447). However, NAFLD fibrosis score could not be calculated because our database did not provide the information about serum albumin level. The mean level of APRI score was 0.29 while the median value of BARD score was 2. The relationship between serum zinc level and APRI score or BARD score was not stronger than that between zinc and FIB-4 index (correlation coefficients r=-0.191, p=0.117 in ARPI score; r=-0.120, p=0.039 in BARD score).

When we assessed the risk factors for significant fibrosis (FIB-4 ≥1.3) (Table 2), we did not include ‘age’ in the multivariable because FIB-4 included ‘age’. Thus, we included sex, BMI, LDL level, r-GTP level, diabetes, and zinc level in the multivariable analysis. We added these considerations in the discussion section of the revised manuscript.

(page 15, line 20)

“We also calculated non-invasive fibrosis markers besides FIB-4 index: (i) AST to Platelet Ratio (ARPI) score =100 × (AST / upper limit of normal) / platelet count (×10^9^/L)[36]; (ii) BARD score = (BMI ≥ 28 = 1 point, AST to ALT ratio ≥ 0.8 = 2 points, diabetes = 1 point), scale 0–4.[37] However, NAFLD fibrosis score could not be calculated because our database did not provide the information about serum albumin level. The mean level of APRI score was 0.29 while the median value of BARD score was 2. The relationship between serum zinc level and APRI score or BARD score was not stronger than that between zinc and FIB-4 index (correlation coefficients *r*=-0.191, *p*=0.117 in ARPI score; *r*=-0.120, *p*=0.039 in BARD score).”

-are there any data of echography or fibroscan in the patient group with NAFLD?

: Thank you for your comment. The survey of this study did not include ultrasonography or transient elastography. The best way to evaluate hepatic fibrosis in the non-invasive manner is transient elastography or magnetic resonance elastography. This study was conducted based on a nationally representative survey complex, stratified, multistage, and probability cluster sampling of the noninstitutionalized population (the Korea National Health and Nutritional Examination Survey 2010). A total of 8,958 participants completed the survey. Therefore, it is difficult to be checked with transient elastography or magnetic resonance elastography in terms of the cost. In this study, we evaluated NAFLD (NAFLD liver fat score) and hepatic fibrosis (FIB-4) using commonly used blood tests. We added this limitation in the discussion section of the revised manuscript.

(page 15, line 16)

“The survey of this study did not include other noninvasive studies such as ultrasonography, transient elastography, and magnetic resonance elastography. If the study could check the noninvasive imaging studies, the diagnostic accuracy of hepatic fibrosis would be improved because the diagnostic accuracy of FIB-4 is a little low compared to that of transient elastography or magnetic elastrography (area under the receiver operating characteristics for significant liver fibrosis: FIB-4 0.75, transient elastography 0.83, and magnetic resonance elastography 0.88) [33].”

-is there any recommendation about Zinc intake of supplementation that can be given to patients with NAFLD?

→Thank you for your comments. The KNHANES provided information about the intake of Zinc-rich foods such as meat, shellfish, beans, seeds, nuts, dairy foods, eggs, and whole grain. But it did not provide information about oral Zn intake. But, several previous studies provided the relationship of oral Zn intake and NAFLD. We mentioned this comments in Discussion section as followed:

(page 16, line 21)

“Although the KNHANES provided information about the intake of these foods, it did not provide information about oral Zn intake. Previous studies reported that oral Zn intake was decreased in patients with NAFLD. Zolfaghari *et al.* showed that oral Zn intake in patients with NAFLD was significantly lower than that in normal healthy controls [35]. Toshimitsu *et al.* revealed that NASH patients had lower Zn intake compared to patients with fatty liver (simple steatosis) [36].”

Reviewer #3: In this study, the authors show that low blood zinc level is associated with higher FIB-4 score in a population of NAFLD-suspected patients (based on NAFLD liver fat score).

Major comments:

- The authors mention in the discussion that “data on NAFLD are limited” (page 12) without giving any reference. However, another study is available with the same observation based on histological results (Takanori et al. PMID 31688305), accepted one year ago. The authors should add this reference and discuss it.

: Thank the reviewer for pointing this out. We described the previous researches for the relationship between Zn and NAFLD in the introduction and discussion sections of the revised manuscript. Kosari et at. also reported the relationship between Zn level and the hepatic inflammation proved by histologic finding. Takanori el al. showed the progression of liver fibrosis, but not inflammation, is associated with lower serum Zn levels in biopsy-proven NAFLD patients. We added these contents in the ‘introduction’ and ‘discussion’ sections of the revised manuscript.

(page 4, line 6) – introduction section

“Recently, the relationship between Zinc and hepatic fibrosis was revealed in patients with biopsy-proven NAFLD.[12] (Takanori et al. PMID 31688305) However, liver biopsy is not widely used as a routine screening tool to detect or monitor fibrosis progression in NAFLD because of its invasiveness and the cost. The present study aimed to examine the role of Zn in patients with NAFLD using a nationally representative database in Korea. We investigated the effect of serum Zn level on hepatic fibrosis represented by simple and noninvasive blood-based biomarker in patients with NAFLD.”

(page 14, line 11) – discussion section

“Kosari *et al*. reported that serum Zn level was significantly lower in moderate and severe lobular hepatic inflammation than in mild lobular hepatic inflammation in patients with NASH although enrolled patients were younger (mean age: 36 years) than other studies and the study excluded cirrhotic patients [32]. Takanori *et al.* showed that the progression of fibrosis , but not the severity of hepatic inflammation, is associated with lower levels of serum Zn in patients with biopsy-proven NAFLD.[12] (Takanori et al. PMID 31688305) There are some differences between studies about race, geography and dietary habits which affects to amount of Zn intake. However, these studies showed a similar tendency in terms of the relationship between low Zn level and the progression of hepatic inflammation in patients with NAFLD. The present study was conducted for reliable and representative participants (KNHANES) and used the FIB-4 index which is objective and well-studied for a noninvasive estimate of liver fibrosis.”

- The criteria used for both NAFLD diagnosis and fibrosis are based on simple non-invasive scores. This should be clearly mentioned in the discussion. Other better non-invasive diagnostic tools exist such as transient elastography or MRI (for the evaluation of both steatosis and fibrosis).

-> We appreciate your valuable comment. The best way to evaluate hepatic steatosis or fibrosis in the non-invasive manner is transient elastography or magnetic resonance elastography. Unfortunately, the study did not check those exams. This study was conducted based on a nationally representative survey complex, stratified, multistage, and probability cluster sampling of the noninstitutionalized population (the Korea National Health and Nutritional Examination Survey 2010). A total of 8,958 participants completed the survey. Therefore, it is difficult to be checked with transient elastography or magnetic resonance elastography in terms of the cost. Instead, this study evaluated NAFLD (NAFLD liver fat score) and hepatic fibrosis (FIB-4) using commonly used blood tests. As you commented, the diagnostic accuracy of FIB-4 is a little low compared to that of transient elastography or magnetic elastrography (AUROC for significant liver fibrosis: FIB-4 0.75, transient elastography 0.83, and magnetic resonance elastography 0.88) (hepatology 2017;66:1486-1501
). We added this limitation in the revised manuscript.

Page 15, line 22

“The survey of this study did not include other noninvasive studies such as ultrasonography, transient elastography, and magnetic resonance elastography. If the study could check the noninvasive imaging studies, the diagnostic accuracy of hepatic fibrosis would be improved because the diagnostic accuracy of FIB-4 is a little low compared to that of transient elastography or magnetic elastrography (area under the receiver operating characteristics for significant liver fibrosis: FIB-4 0.75, transient elastography 0.83, and magnetic resonance elastography 0.88).[34]”

- The Pearson correlation (between FIB-4 and Zn) is weak (-0.120). There is no figure with the graph. The authors should add it as a scatterplot, because it is the main message of the paper.

: We appreciate your valuable comment. According to your comment, we analyzed the correlation between serum zinc and FIB-4 index adjusting potential confounding factors (gender, BMI, r-GTP, fasting glucose level, triglyceride, LDL cholesterol, HDL cholesterol, diabetes mellitus, and vitamin D level). The partial correlation coefficients (*r*) adjusted for gender, BMI, r-GTP, fasting glucose level, triglyceride, LDL cholesterol, HDL cholesterol, diabetes mellitus, and vitamin D level between serum zinc level and FIB-4 index is *r*= -0.177 with P=0.003. We added these findings in the methods and results sections with Figure 2.

Figure 2. The relationship between serum zinc level and hepatic fibrosis (FIB-4 index) in subjects with NAFLD

“*r*” is Partial correlation coefficient adjusting gender, body mass index, gamma glutamyl transferase, fasting glucose level, triglyceride, low density lipoprotein cholesterol, high density lipoprotein cholesterol, diabetes mellitus, and vitamin D level.

Abbreviations: NAFLD, nonalcoholic fatty liver disease; FIB-4, fibrosis-4

(page 7, line 10) – methods section

“Partial correlation analysis between serum Zn level and FIB-4 index was done adjusting potential confounding factors which could affect the relationship.”

(page 9-10, line 16-4)

“We analyzed the correlation between serum zinc and FIB-4 index adjusting potential confounding factors. The partial correlation coefficients (r) adjusted for gender, BMI, GGT, fasting glucose level, triglyceride, LDL cholesterol, HDL cholesterol, diabetes mellitus, and vitamin D level between serum zinc level and FIB-4 index is *r*= -0.177 with *p*=0.003.”

- The reason why the authors included only 6 variables in the multivariate analysis is not clear. It is true that looking for a correlation with the FIB-4 score exclude the evaluation of age, ALT, AST which are usual parameters associated with fibrosis (probably better than zinc levels). Furthermore, BMI (mentioned as included in the methods but no result available) or waist circumference should probably be included.

-> We appreciate your valuable comment. The multivariable analysis was performed as follows in reference to ‘Multivariable Analysis: A Practical Guide for Clinicians and Public Health Researchers 3rd edition’ (2011; Mitchell H. Katz: Cambridge University Press). First, we included in the multivariable analysis any variable that was associated with the outcome at a P-value of <0.10, regardless of whether or not the variable was associated with the risk factor. Second, for multivariable logistic regression analysis, the subjects of analysis should have at least ten outcomes for each independent variable in the multivariable model. Third, to consider the multicollinearity of the variables, the methods of variable selection such as forward selection or backward elimination are used in multivariable analysis model. We used the forward conditional stepwise method (in SPSS 18.0) in this study.

Considering the above-mentioned points, we performed the multivariable logistic regression analysis in this study. In the univariable analysis, sex, BMI, LDL level, r-GTP level, diabetes, and zinc level were statistically significant (p<0.10). In this study, patients with significant fibrosis (FIB-4 >1.3) were reported in 62 patients. Therefore, we selected six variables in multivariate analysis (62 patients/10 = 6 variables). We performed the multivariable analysis with the forward conditional stepwise method and by six variables (sex, BMI, LDL level, r-GTP level, diabetes, and zinc level) which were selected by the univariable analysis. Finally, sex, diabetes and zinc level were the independent risk factors for significant fibrosis after multivariable analysis using six variables. We added these contents in the methods and results sections with Table 2 of the revised manuscript.

(page 7, line 14) – methods section

“Multivariable analysis was done in the following manners. First, we included in the multivariable analysis any variable that was associated with the outcome at a P-value of <0.10, regardless of whether or not the variable was associated with the risk factor. Second, for multivariable logistic regression analysis, the subjects of analysis should have at least ten outcomes for each independent variable in the multivariable model. Third, multivariable analysis was performed using a forward conditional stepwise procedure to avoid multicollinearity. Thus, we included sex, BMI, LDL level, GGT level, diabetes mellitus, and zinc level in the multivariable analysis. We excluded age in the multivariable analysis because the dependent variable (FIB-4) included age.”

(page 7, line 14) – methods section

“The multivariable analysis was done with the forward conditional stepwise method and by six variables (sex, BMI, LDL cholesterol, GGT, diabetes mellitus, and serum zinc level) which were selected by the univariable analysis.”

Minor comments:

- Numerous similar data are presented in the text and in the Table 1 (redundant information).

: As you commented, we truncated those sentences in the revised manuscript.

(page 8, line 5)

“The baseline characteristics of subjects with NAFLD are presented in Table 1. The mean age of the subjects was 49.5 years. The proportion of men was 65.7% (197/300). The mean body mass index (BMI) was 26.2 kg/m^2^. The mean AST, ALT, and GGT levels were 27.6 U/L, 33.8 U/L, and 52.0 U/L, respectively. The proportion of patients with diabetes mellitus (DM) was 21.3% (64/300). The mean FIB-4 index was 0.99 ± 0.60, and the mean serum Zn level was 139.8 ± 29.9 µg/dL.

The comparison of clinical characteristics was conducted between subjects with high Zn level (≥140 µg/dL) and low Zn level (<140 µg/dL). Subjects with low Zn level were older than subjects with high Zn level (51.0 vs 47.7 years, *p*=0.045). The proportion of men was higher in subjects with low Zn level than in subjects with high Zn level (73.3% vs 59.4%, *p*=0.011). There were no differences in HOMA-IR value; NAFLD liver fat score; and presence of DM between the two groups. The serum 25(OH)D level was significantly higher in subjects with high Zn level than in subjects with low Zn level (19.3 vs 17.2 ng/mL, *p*=0.006). There was a significant difference in FIB-4 index between the two groups. The FIB-4 index was higher in subjects with low Zn level than in subjects with high Zn level (1.07 vs 0.89, *p*=0.008).”

- The HOMA-IR is the same in the two groups (high or low zinc level). Is the HOMA-IR available in all patients?

:Yes, all participants totally examined the fasting glucose and insulin level after 12hr of fasting.

- What is the definition of diabetes mellitus? treatment for hyperglycemia? Blood glucose > 126mg/dL?

: In this study, diabetes mellitus was defined as fasting blood glucose ≥ 126mg/dL or treated with anti-diabetic drug. We added this definition in the methods section of the revised manuscript.

(page 6, line 6)

“Diabetes mellitus was defined as fasting blood glucose ≥ 126mg/dL or treated with anti-diabetic drug.”

- Was the HOMA-IR calculated/available also in treated diabetic patients? Under treatment?

: Yes. The KNHANES provided information about fasting glucose and insulin level in all participants regardless of treatment status of diabetic patients.

- Data are available on the daily micronutrients intake in control, NAFLD and NASH patients (for example Musso et al. Hepatology 2003). Are there data on zinc intake?

: Thank you for your comment. The KNHANES provided information about the intake of Zinc-rich foods such as meat, shellfish, beans, seeds, nuts, dairy foods, eggs, and whole grain. But it did not provide information about oral Zn intake. But, several previous studies provided the relationship of oral Zn intake and NAFLD. We mentioned this comments in Discussion section as followed:

(page 16, line 21)

“Although the KNHANES provided information about the intake of these foods, it did not provide information about oral Zn intake. Previous studies reported that oral Zn intake was decreased in patients with NAFLD. Zolfaghari *et al.* showed that oral Zn intake in patients with NAFLD was significantly lower than that in normal healthy controls [35]. Toshimitsu *et al.* revealed that NASH patients had lower Zn intake compared to patients with fatty liver (simple steatosis) [36].”

- Another study with histology (Kosari et al.) is presented in the references. Histological results are also available in this study. However, in this study, there was no correlation between fibrosis and zinc levels but with portal inflammation. This should be discussed.

: Thank you for your comment. We described the previous researches for the relationship between Zn and NAFLD in the discussion section of the revised manuscript. Kosari et at. also reported the relationship between Zn level and the hepatic inflammation proved by histologic finding. Takanori el al. showed the progression of liver fibrosis, but not inflammation, is associated with lower serum Zn levels in biopsy-proven NAFLD patients. We added these contents in the discussion section of the revised manuscript.

(page 14, line 11)

“Kosari *et al*. reported that serum Zn level was significantly lower in moderate and severe lobular hepatic inflammation than in mild lobular hepatic inflammation in patients with NASH although enrolled patients were younger (mean age: 36 years) than other studies and the study excluded cirrhotic patients [32]. Takanori *et al.* showed that the progression of fibrosis , but not the severity of hepatic inflammation, is associated with lower levels of serum Zn in patients with biopsy-proven NAFLD.[12] (Takanori et al. PMID 31688305) There are some differences between studies about race, geography and dietary habits which affects to amount of Zn intake. However, these studies showed a similar tendency in terms of the relationship between low Zn level and the progression of hepatic inflammation in patients with NAFLD. The present study was conducted for reliable and representative participants (KNHANES) and used the FIB-4 index which is objective and well-studied for a noninvasive estimate of liver fibrosis.”
